# Exploring fathers’ experiences of caring for a child with complex care needs through ethnography and arts-based methodologies

**DOI:** 10.1186/s12887-024-04567-8

**Published:** 2024-02-02

**Authors:** Roberta L. Woodgate, Miriam Gonzalez, Jacquie D. Ripat, Marie Edwards, Gina Rempel

**Affiliations:** 1 College of Nursing, Rady Faculty of Health Sciences, University of Manitoba, 89 Curry Place, Winnipeg, MB R3T 2N2 Canada; 2 College of Rehabilitation Sciences, Department of Occupational Therapy, Rady Faculty of Health Sciences, University of Manitoba, R215-771 McDermot Avenue, Winnipeg, MB R3E 0T6 Canada; 3 Department of Pediatrics and Child Health, Rady Faculty of Health Sciences, CE-208 Children’s Hospital, Health Sciences Centre, Max Rady College of Medicine, University of Manitoba, 840 Sherbrook Street, Winnipeg, MB R3A 1S1 Canada

**Keywords:** Fathers, Children, Complex care needs, Ethnography, Arts-based methods, Canada

## Abstract

**Background:**

Although the number of children living with complex care needs (CCN) is increasing worldwide, there is limited data on the experience of fathers caring for children with CCN. This paper reports on findings specific to fathers’ experiences of caring for their child with CCN and highlights recommendations provided for parents of children with CCN, service providers, and policymakers. The findings emerged from a larger study designed to examine how Canadian families of children with CCN participate in society.

**Methods:**

We used the qualitative research approach of ethnography and arts-based methodologies (ecomaps and photovoice) as well as purposive and snowball sampling techniques. Four parents were engaged as advisors and twenty-nine fathers participated in interviews (all were married or in a relationship; age range of 28 to 55 years). In line with an ethnographic approach, data analysis involved several iterative steps including comparing data from the first, second, and third set of interviews and refining themes.

**Results:**

One overarching theme, *striving to be there for the child with CCN*, was identified. Five supporting themes further exemplified how fathers strived to be there for their child: 1) contributing to the parental team through various roles; 2) building accessibility through adaptation; 3) engaging in activities with the child; 4) expressing admiration and pride in their children; and 5) meaning making. Recommendations for parents included making and nurturing connections and asking for help while recommendations for healthcare and social service providers included communicating authentically with families and listening to parents. Fathers also indicated that leadership and funding for programs of families of children with CCN should be priorities for policymakers.

**Conclusions:**

In addition to documenting fathers’ active involvement in their child’s care and development, our findings provide new insights into how fathers make participation in everyday life accessible and inclusive for their children. Study findings also point to 1) priority areas for policymakers (e.g., accessible physical environments); 2) factors that are critical for fostering collaborative care teams with fathers; and 3) the need for complex care teams in the adult health care system. Implications for those providing psychosocial support for these families are noted as well as knowledge gaps worthy of future exploration such as the role of diversity or intersectionality in fathering children with CCN.

**Supplementary Information:**

The online version contains supplementary material available at 10.1186/s12887-024-04567-8.

## Background

The number of children with complex care needs (CCN) is increasing worldwide [1–4]. Children with complex care needs are those with one or multiple chronic health conditions and/or disability(ies), who often depend on assistive or medical technology, and require intensive use of healthcare services as well as social and educational supports [5–15]. Although various terms are used in the literature to refer to this population of children (e.g., children with life-limiting conditions, children with chronic illness, and children with medical complexity) [16, 17], we use ‘children with complex care needs’ as this term shifts the emphasis from labeling the child based on a specific health condition to the support needed from others or society [17, 18]. In Canada, a national study identified 97, 561 children and youth with complex care needs in 2015–2016, a rate of 948 per 100,000 children and youth [19, 20]. As a result of advances in medical care and technology, more children with CCN survive into adulthood [21–23] and the ongoing, complex care needed by these children is being provided by primary caregivers in the home [7, 17, 24–26].

With changes in society’s view of fatherhood from seeing fathers as mere wage earners to active participants in their children’s lives, research in the last few decades has documented fathers becoming more involved in family life and the care and support of their children [27]. Although various theoretical conceptualizations of father involvement have been proposed [28–31], one definition highlights that both the quantity of engagement with the child and the quality of the father-child relationship (e.g., warmth, responsiveness to child) are important aspects of father involvement [32]. Father involvement in child-rearing has been associated with positive developmental, cognitive, and socioemotional child outcomes [33–36]. Emerging evidence also points to the positive impacts of father involvement for families of children with chronic health conditions. Increased father involvement in these families has been associated with better adherence to treatment [36–38], positive parent-reported quality of life scores and health-related outcomes for the child [38, 39], fewer self-reported maternal psychiatric symptoms [40], better marital functioning [40], less negative impacts of the illness on the family [38], and better overall family functioning [38, 40–42]. Despite these positive impacts and increases in father involvement in child-rearing activities, there is a paucity of research on fathers’ experiences and involvement in caring for a child with complex care needs [27, 36, 41]. Research on these families has focused on service delivery and care coordination [21, 43]. Further, the growing body of research about the experiences of parents of children with CCN has focused primarily on mothers’ perspectives [16, 17, 26, 44–46]. For instance, of 17 studies included in a meta-ethnography of the experiences of parents of children with life-limiting conditions, only one study explored the experience of fathers [46].

There is also limited data about the experiences of Canadian fathers of children with complex care needs. In a 2021 systematic review of qualitative studies exploring the experiences of fathers of children with life-limiting conditions, only 3 of the 30 studies included in the review were conducted in Canada [47–50]. Further, all three studies explored the experiences of fathers of children with cancer and only one study was conducted in the last ten years. Similarly, in a 2023 systematic review of fathers’ experiences of caring for a child with a chronic illness, only one of the ten studies included in the review involved Canadian fathers [41]. However, this study involved data collection in multiple countries (United States, India, Italy, United Kingdom, and Venezuela) and the Canadian sample consisted of five fathers involved in the complex management of their child’s type 1 diabetes [51].

Recognizing that health and social systems vary amongst countries, there is a critical need for Canadian-specific data about fathers’ experiences of caring for a child with a range of complex care needs. The dearth of Canadian data in this area suggests that: 1) fathers’ experiences are not well documented or understood; 2) current clinical practice with fathers is primarily informed by mothers’ perspectives [52]; and 3) there is a need for data to inform clinical practice with fathers as well as policy and programming designed to support fathers in their caregiving role. The purpose of this paper is to detail Canadian fathers’ experiences of caring for their child with CCN and to highlight recommendations they provided for other parents of children with CCN, healthcare and social service providers, and policymakers. The findings emerged from a larger study designed to examine how families of children with CCN participate in society [12–15]. To our knowledge, this is the first Canadian study to use an ethnographic approach and arts-based methodologies to attain a comprehensive understanding of fathers’ experiences in this context.

## Methods

### Study design

We used an ethnographic qualitative research approach to provide a detailed account of families’ lives and roles as carers of children with complex care needs. We chose ethnography as this approach: 1) supports the view that individuals are competent reporters of their own experiences; 2) puts families’ views at the centre of analysis; 3) ensures that the research works for families rather than on them; and 4) facilitates obtaining a thick description of families’ social worlds and experiences [53–55]. Our ethnographic approach included collecting data over 3 years using in-depth interviews, photographs, and ecomaps. We used triangulation (photos, ecomaps, and interview data), in-depth analysis including comparing the data obtained, reflexivity, and thick description to provide a comprehensive and detailed account of our findings [53–55]. Arts-based methodologies (i.e., ecomaps and photovoice) were used to facilitate discussion and provide creative ways for parents to share their stories. Rooted in clinical family nursing practice and family therapy, an ecomap is a graphic portrayal of social relationships or networks between individuals, families, places, and settings [56]. Ecomaps facilitate understanding the nature of the bonds and the degree of attachment that individuals experience [56]. The participatory research method of photovoice was used to engage participants (i.e., document/reflect on their experiences) by asking them to take photographs representative of their experiences and providing captions for the images [57].

### Participants and recruitment

Families of children with complex care needs were recruited from a primary integrated health and social services community program located in a major city in Canada. Families who accessed this program, had a child with CCN, and requested one or more of the services offered through the program (respite, preschool therapy, child development services, behaviour psychology services, recreation services, and/or assistance with extraordinary costs of caring for a child with CCN) could participate. We used a combination of purposive and snowball sampling techniques to be inclusive of the experiences of families from diverse backgrounds. Recruitment ended once theoretical saturation was achieved.

In total, 68 parents (29 fathers) from 40 families (6 families had no fathers) participated in the study. Of the 68 parents, 58 were married or in a relationship (29 fathers were married or in a relationship). The remaining 5 fathers from the 40 families decided not to participate. No information was collected as to their decision not to participate. The age of the fathers ranged from 28 to 55 years (see Table 1). Nearly 28% (*n* = 8) of fathers reported an annual income of $50,000 or less, 38% (*n* = 11) had a high school degree or less, and 2 fathers were in same-sex relationships. Fathers described their child’s condition as complex given the ongoing medical and/or pharmacological treatment needed as well as the condition’s severity, rarity, and comorbidities. The children ranged in age from 6 months to 26 years (mean age of 10 years) and the majority were male (*n* = 23). Their primary diagnoses included cerebral palsy, developmental disorders (e.g., global developmental delay), terminal cancer, seizure disorders, chronic lung disease, congenital disorders, and genetic disorders (e.g., trisomy 21).

**Table 1:** Fathers’ characteristics

Variable	*N* = 29
**Age (*n* = 26)**	
20–29 years	1
30–39 years	4
40–49 years	18
50–59 years	3
**Ethnicity (*n* = 25)**	
European descent	21
Indigenous	1
Other (including Asian, African, Jewish, Arabic, Canadian)	3
**Education (*n* = 26)**	
Grade 8 or less	1
Some high school	7
High school diploma	3
Some college	5
College degree	2
Some university	4
Bachelor’s degree	3
Master’s degree	1
PhD	0
**Household Income (*n* = 28)**	
Less than $10,000 CDN	1
$10,000–20,000	1
$21,000–30,000	2
$31,000–40,000	2
$41,000–50,000	2
$51,000–60,000	2
$61,000–70,000	2
$71,000–80,000	2
$81,000–90,000	1
$91,000–100,000	7
More than $100,000	6
**Relationship Type (*n* = 29)**	
Heterosexual	27
Same sex	2

### Data collection

Prior to data collection, the study’s research assistant (university educated) provided potential participants information about the study and about the principal investigator and first author (e.g., a Distinguished Professor and researcher who conducts and engages youth and families in multi-methods and qualitative arts-based research to improve their well-being). Parents who agreed to participate provided written informed consent at the beginning of the study. Data collection took place over three years (2009–2011) and involved interviewing parents at three time points: at the beginning, midway, and towards the end of the study. Thus, an on-going consent process was in place that involved obtaining verbal consent at the two additional data collection time points.

Parents completed a demographic form and then took part in semi-structured, open-ended interviews designed to explore parents’ experiences of raising a child with CCN. When both parents were available to be interviewed, they were interviewed together. Otherwise, fathers were interviewed separately. The interviews were conducted by a research assistant who received training and was supervised by the first author. For the first interviews, the interview guide included questions about raising a child with CCN. For example, parents were asked to describe what a typical day was like for them, discuss how things were different since having to care for their child, and outline challenges encountered. For the second and third interviews, questions based on the emerging data analysis were added to the interview guides. Although an interview guide was used for each of the interviews, the open-ended nature of the interviews gave parents the opportunity to speak about areas they considered important and/or areas not anticipated by the researchers [58].

To complement the first interview and facilitate discussion, parents were asked to draw an ecomap. They were asked to draw: 1) a circle that represented themselves; 2) additional circles representing people, activities, and places that are part of their lives; 3) lines between the circles to indicate connections between each person, activity, and place; and 4) different types of lines to represent different types of connection (e.g., thick line represented a strong connection). During the second and third interviews, parents were asked to reflect on any changes to their ecomaps. At the end of the first interview, the photovoice method was explained to parents. They were given digital cameras and asked to take photos of places, events, objects, and people (with their permission) that represented their everyday life.

During the second interview session, parents were asked to speak about the photographs by means of the SHOWED method [59]. This method encourages discussion on the meaning of the photos and involves asking parents to describe what is happening in the photos and to explain how the photos relate to their lives [59]. Parents were also asked follow-up questions based on their first interview and to speak about any changes to their caregiving experience since their last interview. During the third interview, parents were asked whether participating in everyday life had changed since taking part in the study and whether participation in the study had resulted in any new reflections about their everyday life. Parents received an honorarium in the form of a gift card in appreciation for their participation.

Parents from 29 families participated in the second interview and parents from 20 families took part in all three interview sessions. Interviews were conducted in parents’ homes, lasted from 90 to 180 min, and were digitally recorded and transcribed verbatim. Field notes were recorded to describe the interview context (e.g., parents’ nonverbal behaviour, communication processes, interviewers’ perceptions of the interview).

### Data analysis

Univariate descriptive statistics were used to explore participant demographics. Analysis of the qualitative data occurred concurrently with data collection. All data emerging from interviews, ecomaps, photographs, and field notes informed the analysis and were organized using the software Microsoft Word. In line with an ethnographic approach, data analysis involved several iterative steps [54]: 1) isolating items or patterns, organizing these patterns, and identifying attributes for each; 2) identifying relationships among the patterns to create themes; 3) comparing data from the first set of interviews with the second and third set of interviews; and 4) refining themes. The first author and the second author (a PhD trained mixed methods researcher with training and experience conducting qualitative research) collaborated on all steps and discrepancies were resolved through discussion. Methods to enhance methodological rigour and trustworthiness included prolonged engagement with participants and data, careful line-by-line analysis of the transcripts, detailed memo writing, member checking (e.g., preliminary interpretations were discussed with parents to validate the emerging themes), data triangulation (e.g., interviews, ecomaps, photovoice), and providing rich description of the findings [60].

### Family engagement in this study

Four parents (two mothers and two fathers) of children with complex care needs were research partners and members of this project’s Family Advisory Committee (FAC). Following funding acquisition for the project, the parent partners were recruited through the first author. The first author discussed with parents how they wished to be involved prior to engagement in the FAC. Parent partners participated in meetings, data analysis (reviewed and provided input on themes), and the knowledge translation phase (provided ideas for knowledge translation products that included videos created for dissemination purposes). They received an honorarium for their participation.

## Results

We identified one overarching theme and five supporting themes. The overarching theme is striving to be there for the child with CCN. This theme refers to fathers making the time to exercise their parenting role, be a part of the child’s life, and support and help the child. One characteristic of ‘striving to be there for the child’ involved hands-on father involvement ranging from engaging in caring activities to participating in activities with their children:

“I’ll get up in the morning and I’m responsible for getting the kids ready. So that means making sure “K” [other child] gets up and then for “J” because she’s full needs, you have to get her up and wash her face, brush her hair, get her dressed, then get her into the kitchen to have breakfast. You make her breakfast. She needs her hearing aids put in. Her glasses put on. She needs her teeth brushed afterwards. Make sure that she goes to the toilet before the bus picks her up. The bus comes, you get her on the bus.” (F10)

Another father shared:

“And we just recently got a bike for her. And I pedal the bike and she rides. She’s in front of me and the wheelchair’s got these handlebars, which is how we steer the bike. And if you want to stop and detach the wheelchair, one latch takes it off, then she goes wheeling around in the wheelchair.” (F17)

Another characteristic of ‘striving to be there for the child’ involved being there with and for the child in mind and spirit. This included being physically present for the child, providing nurturance (e.g., in everyday life, in times of distress), and making meaning of their fathering a child with complex care needs. One father shared:

“She was having a rough night, so I had her in a cuddle while she was you know getting her chemo and the Kingpin was on at 3:00 in the morning and we were watching it and we were always laughing. She was laughing then because I was laughing and yeah.” (F17).

**Fig. 1:**
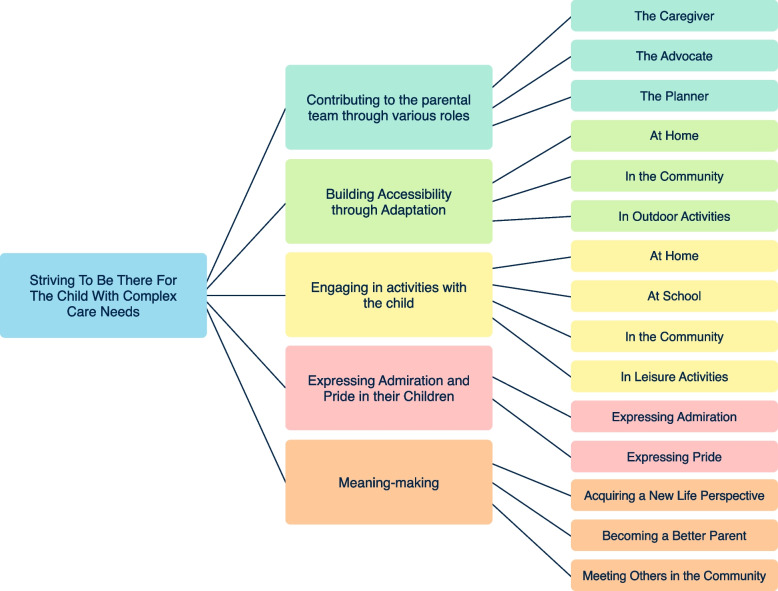
Fathering a Child with Complex Care Needs (CCN): Overarching Theme, Themes, and Subthemes

The five supporting themes further exemplify how fathers strived to be there for their child: 1) contributing to the parental team through various roles; 2) building accessibility through adaptation; 3) engaging in activities with the child; 4) expressing admiration and pride in their children; and 5) meaning making (see Fig. 1. Fathering a Child with Complex Care Needs (CCN): Overarching Theme, Themes, and Subthemes).

### Theme 1: Contributing to the parental team through various roles

**Fig. 2:**
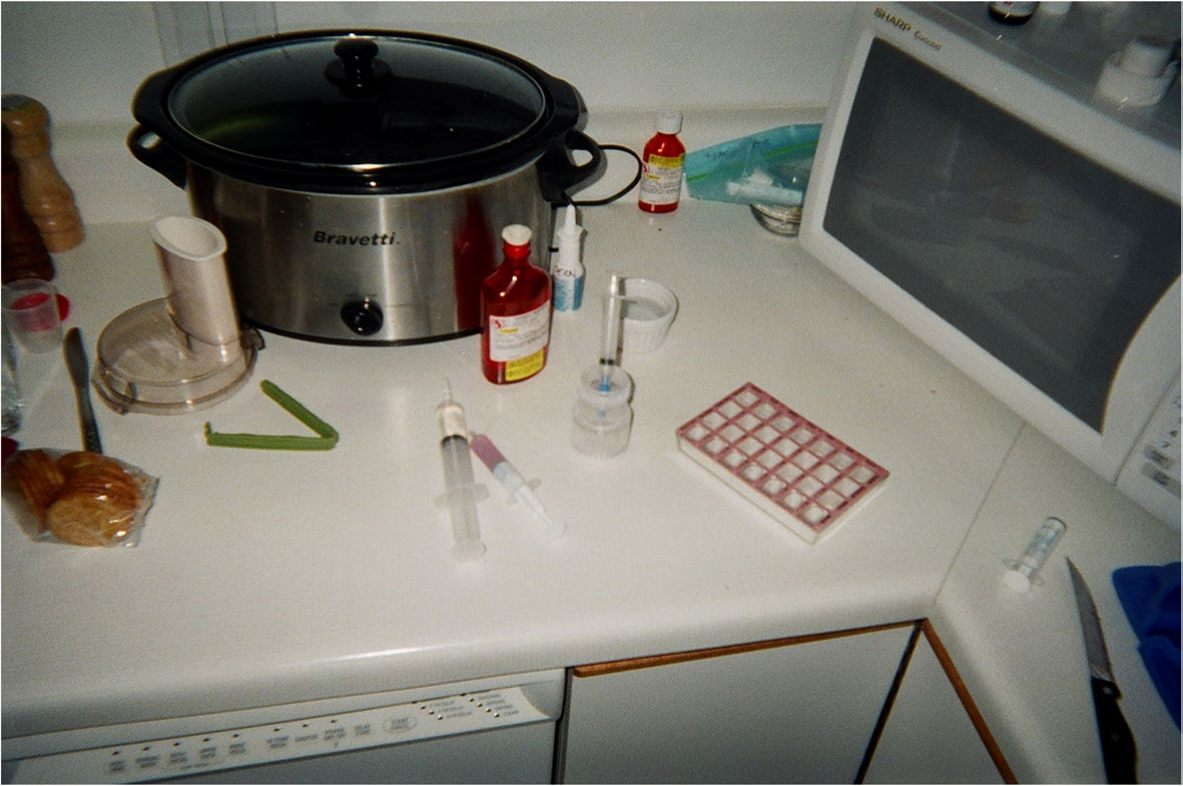
Medication Preparation

Fathers spoke about sharing the responsibility to care for their child with CCN. They described a team-like atmosphere, a ‘tag team’ approach [12], where care was a shared effort with partners, and they never felt alone when providing care. Yet, they spoke about various roles they adopted in the life of their child: caregiver, advocate, and planner. These roles were enacted in the context of a team-like atmosphere. In their role of caregiver, fathers described learning about their child’s condition, medications, and how to care for their child (e.g., providing meals by a tube). A photovoice submission (see Fig. 2 Medication Preparation) shows one aspect of fathers’ caregiving role: preparing the child’s medications. The father noted that preparing the medications was fairly routine but if one medication was forgotten, trouble was imminent.

Fathers also helped with activities of daily living. They spoke about sharing responsibilities such as bathing, dressing, carrying the child, taking them to daycare/school, going to appointments (e.g., doctor, rehabilitation, school appointments) and putting them to bed. One father shared: [about providing meals for his child].

“I learned in the hospital so when I came home, I knew what I was doing…He eats. He just started eating now like solids and stuff. We’re using the tube for his calories because he uses a different milk cause he’s allergic to milk. So, he uses Pregestimil and I just shoot it through [the tube], takes a couple of *minutes*.” *(F4).*

Another father shared he would take his son to medical appointments and conveyed his frustration when clinics would prefer speaking to his wife:

“I was the one taking “D” to most of his appointments for so long. They would phone to talk to “M” [the mom] or you know, “Maybe we should talk to ‘D’s’ mom.” It’s like, you know, get with it. Moms and dads have equal responsibility for the kids now. It’s not 1960 anymore.” (F6).

Many fathers also spoke about being an advocate for their child. They spoke about having to learn to advocate because of their situation, having to push for what their family needed, and described advocating as a full-time job:

“I think the biggest challenge overall on a day-to-day over year-to-year, *a full-*time* job, it’s trying to be “C’s” advocate and trying to get all the programs set up and all the nursing set up, and all the homecare, and all the different programs.” (F17)*

Fathers viewed advocating as very challenging and emotionally draining. Another father described his experience:

“Well, the amount of respite that we required was never enough. And that’s the problem with dealing with these agencies. Instead of having somebody coming and saying ‘Okay, you guys need help. I’m going to get it for you.’ It’s you basically having to scream at the top of your lungs saying, ‘I need some help here. Get it to us now or we’re going to be taking this child and throwing them out of the house.’ And it seems like that’s the only time you get reaction from these people. But it’s only when you start to scream that they start to say: ‘Oh well, yeah okay we can give you some extra stuff’.” (F10).

Fathers reflected on their advocacy experience, expressed concern for families who did not know how to advocate, and reflected on how their advocacy may be perceived by medical professionals:

“I can see the position of someone in the medical community or in the support community, you know, not enough money, not enough people, not enough time. I can put myself in their position and understand how they feel, but by the same token, my concern is my son, not your feelings. So, I find that the really good people in the medical community not only understand that but say that’s what you have to do. They don’t bitch and complain because parents are standing up for their kids because they realize that if they were in your position, that’s exactly what they would do too.” (F6).

Many fathers also assumed the role of planner. This included planning to go to medical appointments, planning to do fun activities, and planning for the future. One father noted there was an extra level of planning involved when it came to his son: *“It would be nice to get away for a weekend without having to plan everything to the nth degree.” (F6).* Future or long-term planning for meeting the child’s future needs (e.g., accommodation needs, suitable longer-term care) was also common. Fathers who had children younger than 18 years of age worried about transitioning out of the pediatric complex care system and what that entailed:

“He is eleven years old. So you know, seven years he’s an adult. There’s a big transition that goes with that. So, plan now for the future because I think we both realize at one point in his life he’s going to end up in a group home or some sort of care facility. If we don’t start planning now, then we’re not going to get to place him in a facility that’s going to give him the best care possible. Some of the facilities out there are for mercy care, are not the places you want to be in.” (F27).

Fathers reflected on these transitions and expressed concern about having to plan in an unfamiliar adult health care system (for those wishing to keep their child at home) or planning transitions to adult hospitals or community facilities. Given this concern, fathers highlighted the need to start planning early.

Theme 2: Building Accessibility through Adaptation This theme refers to fathers making participation in everyday life accessible for their child by making modifications to the physical environment and obtaining or adapting needed technology, devices, or tools. Fathers worked hard to ensure their child with CCN could participate in everyday life at home, in the community, and in outdoor activities. For instance, fathers spoke about making adjustments to the family home and cottage. One father noted: “*I made a whole bunch of changes to the house, you know. You have to have a trampoline in the house for him to bounce on. You got to have the ball pit downstairs.” (F19).* Another father spoke about building the cottage to make it ready for vacations and weekends:

“We’ve got a cottage. We can build it from scratch and put things in. So, as long as the weather cooperates from sometime in May until end of September, we can go up there for vacations and weekends.” (F9)

Some fathers also spoke about ‘adapting or upgrading’ their car (e.g., getting larger vehicles):

“When I started out a Jeep Cherokee and then moved up to a Pathfinder, which had more cubic space inside, and now I’ve got a Durango, which is nice because “D’s” cruiser [an adapted bike] can go right across the back. I don’t have to pull the seats down to put it in there. I can carry the stroller and pick up a load of groceries. He enjoys car rides I think, but certainly even if I get him from daycare and he’s tired or he’s had a seizure, nine times out of ten he’s wide awake in the car looking around watching.” (F6).

**Fig. 3:**
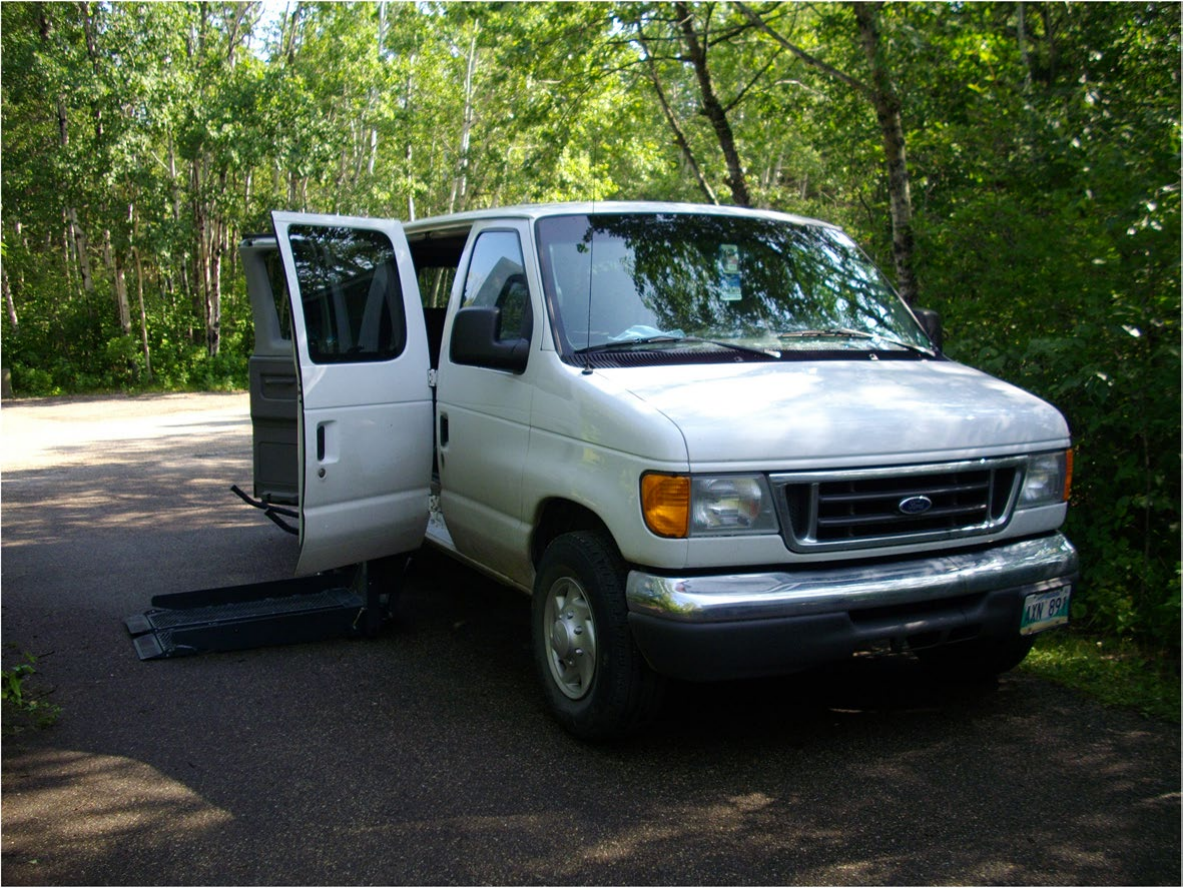
Adapted Truck

This facilitated moving around in the community: to and from daycare, school, hospital, medical appointments, and recreational activities. One photovoice submission (see Fig. 3 Adapted Truck) shows a father’s truck with a wheelchair lift he had leased. He explained the lift made life easier as his daughter could stay in her wheelchair instead of having to get her out of the wheelchair and into the car seat.

Participation in outdoor activities (e.g., camping, bike riding, sailing, boating) was also important to fathers and they relayed how they bought or adapted devices and /or technology to make participation in these activities possible for their child. One father shared he bought a bigger trailer so his child could partake in camping activities:

“We had this pop-up and then the pop-up was getting awkward for her equipment, and she was growing, and her equipment was growing. So, we went and bought ourselves a big trailer and the trailer, it’s like an apartment on wheels I call it. So now she has all the amenities of home. So, we’re not roughing it but we’re still in the campsite. So, we’re doing things, we’re doing things as a family.” (F17)

Another father shared how he made bike riding more accessible for his child:

“Another thing that I did once or twice last year with ‘M’, we wanted to take her for a bike ride. We have a Wike bike trailer for her. It’s a trailer. It attaches to a bike, it’s specifically for special needs. I was mounting her car seat in this thing on cushions and strapping it in so it was safe, just to be able to take her for a bike ride.” (F1)

### Theme 3: Engaging in activities with the child

**Fig. 4:**
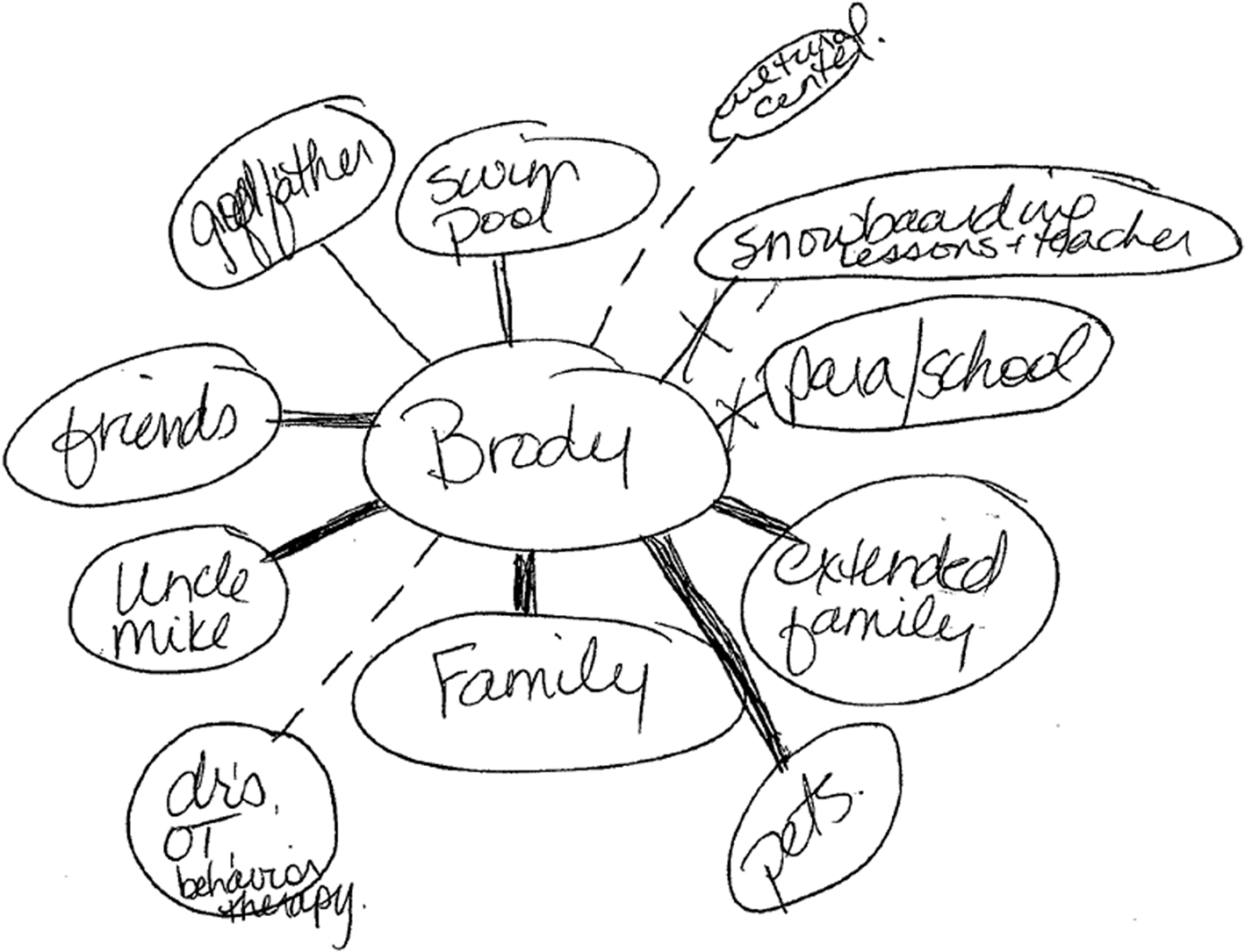
Ecomap

To ensure inclusion and enjoyment by the child, fathers engaged in activities with the child with complex care needs at home, at school, and in the community (e.g., hockey games) including leisure activities. While some of these activities happened in the context of family, most of these activities only involved the father and their child. As reinforced by the ecomap below (Fig. 4 Ecomap) fathers perceived engagement in family, school, and community activities as central to the child’s life.

Fathers reported engaging with the child in home activities such as having meals together or sitting in the backyard. One father shared: *“We do a lot of that [family time]. We have the best times and the most memorable times when it’s just us” (F29).* However, bonding with the child with CCN by spending one-on-one time was very important to fathers. The father of a 15-year-old boy explained:

‘The man cave is in the basement and no women are invited. Quite often, after dinner, I’ll take him down to the men’s zone downstairs and we’ll watch TV or whatever and mostly he’ll just sit on me and read his books and I’ll watch TV, but we spend a little time together.” (F6).

Other home activities that involved spending one-on-one time with the child with CCN included caring for pets and taking the child to music lessons. One father shared how he and his child took care of their pet and how this made the child happy: *“We clean the cages every two days, and he was sitting at the chinchilla cage and just patting the chinchilla. You can see that it just brings a sparkle to his eye.” (F19).*

Taking part in school activities and going to birthday parties also provided socialization opportunities and were important to fathers. Fathers reported enjoying taking their children to these outings:

“I went to the zoo with ‘C’s’ class last summer. That was a lot of fun meeting his friends at school and just hanging around with the other teachers and talking with them. The experience was great. I loved it. So, I’m going to do it again.” (F32)

Another father shared:

“We try to do everything we can with him. There’s nothing that really says we can’t do anything with him. Birthday parties. The beach. Whatever we do, he’s part of it.” (F4)

Taking their children to activities in the community (e.g., sporting events such as baseball or hockey games, amusement parks, special needs events, work functions) was also common. While many fathers noted some activities in the community were not accessible (e.g., lack of accessible spaces), some fathers shared that some venues and events accommodated their children: *“We’re used to taking him to a hockey game. They have special accommodations and information about all the things you can get, not get, all the things that are available” (F19)*. These activities provided a break from the ‘typical’ days of health care scenarios and were enjoyed by all.

Fathers also reported engaging with their children in different kinds of leisure activities**.** This included fishing, sailing, kayaking, and going on vacation. One father shared:

“[the child] caught a fish. Yeah, he loves the boats and actually my sister has a sailboat now, and we’re going to the lake. So, we’re going to be going on that. He’s going to love that. He loves the sand and all that stuff. So, it’s good, you know.” (F32)

Another father described how he went on vacation with his daughter despite other family members’ perceptions that the child could not handle it:

“Me and [child] went to Disney World. And ‘A’ had said: ‘No, [child] can’t handle it, so we’ll pass.” So, I said: ‘Okay I’ll go.’ I went with her, and she thrived. I remember being there. It was 30 above. We were able to sit on the bench when everybody was maybe having an ice cream cone. We’d sit at the bench, do a quick catheter, boom we’re done. We kept her routine up the whole time. She loved it. She had this smile on her face in Disney World. And then at night, the first time we ever heard her- she was laughing in her sleep. She must have had a good dream.” (F17)

### Theme 4: Expressing admiration and pride in their children

Feelings of admiration for their child and feelings of pride in their child were part of the fathering experience. Fathers expressed being impressed with their child’s qualities, happiness, participation in life, accomplishments, and how the child was received by the local community. One father shared:

“I get inspired by ‘B’. First of all, you know, his attitude. When you look at him, he glows. He’s got a phenomenal personality and with the challenges that he has, I’m just amazed on how he does some things. So, I’m inspired at all times.” (F34)

Admiration and pride in their children nurtured their relationships and in turn, reflected strength in the fathers. Fathers also shared stories about how well their children were doing and demonstrated a great deal of pride in how their children were connecting with other people and were supported by the community. One father shared:

“Okay, let me give you one good example of how he relates in a community. They had their high school graduation. It was 270 something people graduated. Everybody went across. ‘M’ was the only one that had an instantaneous standing ovation.” (F24)

**Fig. 5:**
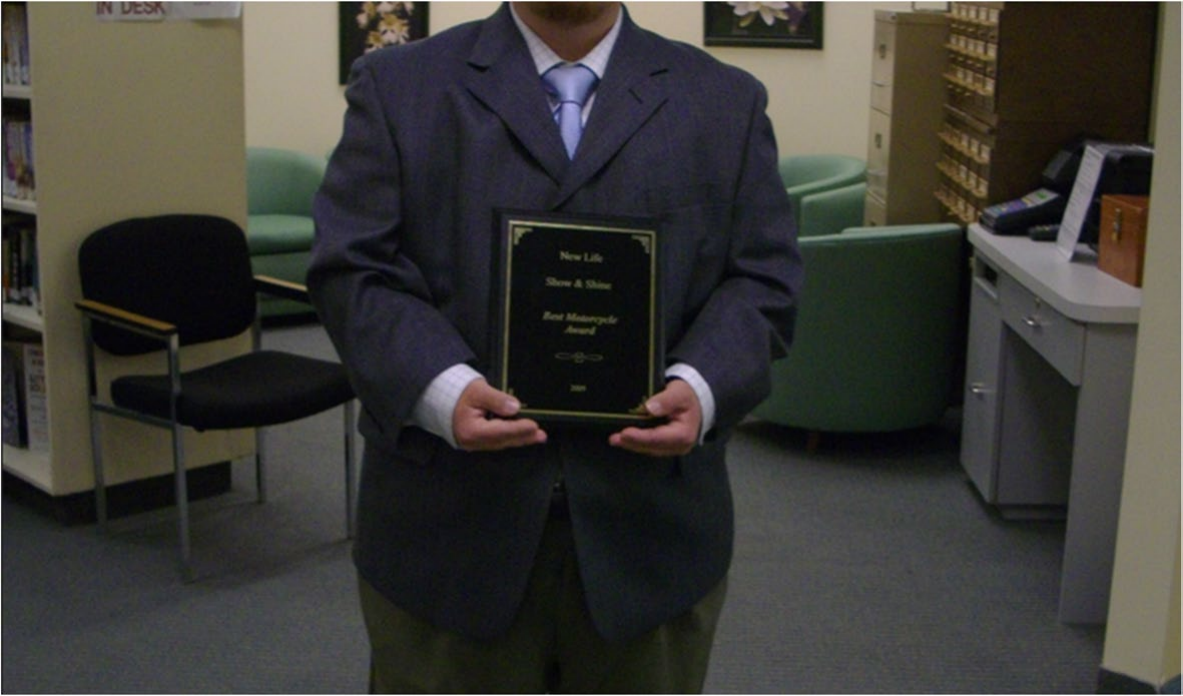
Pride

One father’s photovoice submission (see Fig. 5 Pride) shows his child holding an award received at a church picnic. The father explained the event included a motorcycle show and car show with awards given to the best car and motorcycle. His son entered the contest with his bicycle trike and people voted. This father expressed pride and happiness his son had received the best motorcycle award.

Fathers’ admiration and pride were replenished by reflecting on their children’s achievements:

“He started to learn more and now he’s, you know, learning to his potential for the condition that he’s got. He’s far better than what I thought. They explain it to you like, I don’t know, that he’s only going to be doing certain things and doubt. And he’s doing more than what they told me.” (F4).

### Theme 5: Meaning making

Although fathers acknowledged the stresses of caring for a child with CCN and that they did not wish this on anyone as a learning experience, they experienced meaning-making as they looked for and identified the positives their child with CCN brought to their lives. Fathers noted that this experience brought them unexpected opportunities: acquiring a new life perspective, becoming a better parent, and meeting others in the community.

Fathers reflected on the challenges faced by their children and reported this helped them acquire a new life perspective: maintaining a positive attitude, enjoying every moment with their child, and appreciating life. Keeping a positive attitude often meant learning from their child’s happiness. One father explained: “*This kid’s been through so much and she's always smiling. So why, why, why would we expect any less of ourselves?” (F17).* Fathers also described making the best out of their situation, knowing their situation could be worse, and learning to discern what is important and what is not. One father reflected on how caring for a child with CCN shifted his perspective on what is important in life:

“I think that it has opened a lot, you realize that money’s not the most important thing in life. It’s not your career, I used to be completely career-oriented. Now I realize that as long as I can keep a roof over their, our head and keep them fed and stuff, like that I think it’s good.” (F19)

To fathers, their child with CCN was also a reminder to appreciate life and enjoy every moment they had with them. One father explained:

“I was like okay, that’s the way we’re going to approach it. So, we’ve never approached it as: Oh my god, what are they going to do? What are they going to do? It’s basically, here’s our option: do the chemo and get better and then enjoy all our time with her. We’re going to enjoy whatever moments we have with her. Her life won’t be, she won’t live to seventy. She probably won’t outlive us, but whatever, we’re going to enjoy every moment.” (F17)

Fathers also spoke extensively about how fathering a child with CCN helped them become a better parent. Fathers reported learning to be more open-minded (less judgemental) and accepting of children with CCN and others in general. One father expressed: *“Having a child with a disability like “J”, you learn to accept not only her, but anybody else you come in contact for who they are, whether they have a disability or not, it might be just a personal trait.” (F10).* Some fathers emphasized that fathering a child with CCN also made them more patient and understanding:

**"**I think it teaches you to be more patient. Now if I see a kid in a store screaming and having an outburst I don’t just say: ‘Oh, this kid is spoiled’. Now I think, well you know, this child might have autism. There might be a special need there.” (F19).

Some fathers also expressed that they and their families had learned a lot: their child with CCN had taught them, their partner, and their typically developing children about compassion, caring, understanding, and love. "We've learned a lot [his family]. I mean 'J' has taught us a lot about self-control, about compassion, patience, understanding." (F27).

Finally, fathers in this study reported that fathering a child with CCN had created the opportunity to meet people (e.g., other parents and their children in similar situations, healthcare professionals) and to be part of a community (e.g., philanthropic groups, community organizations or associations, parent groups). However, they expressed this was probably an opportunity other families would not want to have. As one father put it:

**"**We’ve met some nice people. We’ve met people we would never have exposure to. I think of the medical specialists that we’ve dealt with- we’ve met some great people who have helped us out in a lot of ways. I guess those are opportunities to meet these people, but those are probably opportunities that the average family would never want to go to.” (F27).

Some fathers also shared they had the opportunity to help other parents in similar situations by sharing their experiences and knowledge. By getting involved with community organizations they noted they could ‘maybe help change things.’ Fathers also noted that their families (spouse and typically developing children) were also more involved. One father shared:

“We’ve had an opportunity to share our experience with people that are just getting into where we were. We’ve had that opportunity to share and to help and to say you know we’ve been there, you will get through it. And sometimes that’s all they really want to hear, that there is an end to this, or, you know, we will get through it and it gets better.” (F24).

### Recommendations for parents, service providers, and policymakers

Fathers provided recommendations for other parents, healthcare providers, and policymakers.

Making new connections and keeping old connections, asking for help and taking the help, and advocating for the child were the most frequently reported recommendations for other parents. Table 2 below presents the recommendations with representative quotations.

**Table 2:** Fathers’ Recommendations for Parents, Service Providers, and Policymakers

Recommendations	Representative Quotation
**For Parents**	
1. It is all about connections (utterances: 11)	*“Make connections in all the groups that have to do with your child’s disability for sure, but also keep connections in communities at large.” (F18)* *“Try to maintain the contacts you had prior to having your child and, and not let those go and don’t trek into your own little world.” (F10)*
2. Ask for help and Take the help (utterances: 9)	*“And don’t be afraid to ask people for help and don’t be afraid to accept it. I know that goes against a lot of us, I know it did us, but you will need help, just suck it up and take it.” (F18)*
3. Advocate for your child (utterances: 8)	*“Just get ready for the fight. Put aside 50% of your resources and get ready for the fight, cause you’re going to have to. The system is not going to reach out to you. You have to fight for your child.” (F20)*
4. Get involved and Get your child involved (utterances: 7)	*“I would say find something that the child can participate in or you and the whole family can participate in. It gets rid of some of the stress.” (F19)*
5. Don’t neglect yourself or your partner (utterances: 5)	*And then you can’t forget about yourself. It’s very easy to do that and I mean I’m talking about just not the individual but you as a couple. Because it happens so fast you don’t even realize it and instead of being a couple, now you’re individuals that are living in this house, but looking after the same individual.” (F10)*
**For Health & Social Service Providers**	
1. Communicate authentically with families (utterances: 12)	*“Be very forward, don’t hide anything from parents.” (F18)* *“It would be nice if there was something out there that explained everything right away. This is what you’re entitled to, this is what is out there, this is what’s happening. And we have spoken to social workers about that.” (F20)* *“Don’t use those big words when you’re talking to people. I don’t know how you put that, I don’t know – get down to their level and talk to them eye to eye. Tell them straight the, about things, not using the big words and stuff like that.” (F8)*
2. Listen to parents (utterances: 9)	*“Listen to the parents. Yeah, take an extra minute. Don’t rush us out the door.” (F6)*
3. Show that you care (utterances: 7)	*“What I would say makes a difference between a great doctor and an okay doctor or a nurse is just that ability to empathize. I know you can’t put her name in this but that’s what I love about Dr. X. She’s dedicated herself to this. I know she cares, even though I don’t think she really understands exactly what it’s like, but she tries, you know? She tries to kind of understand and you really feel that from her.” (F18)* *“We are not parents that are going in’cause our child has pneumonia and this is the only time my child’s ever going to be in the hospital. We’re in there on a regular basis. We’re involved with the medical community on a regular basis. Don’t forget that. Have a bit more compassion you know? Some people in the medical community don’t have that compassion.” (F17)*
4. Recognize parents as experts on the child (utterances: 4)	*“Trust and believe what the parents are saying. We’re the ones that spend 24/7 with him type of thing. And if we’re telling you that there’s an issue, believe that there’s an issue and understand that there’s an issue.” (F22)*
5. Be proactive (utterances: 4)	*“That’s another thing. Be proactive. The doctor we have now, Dr. “M” is very proactive- asking us, getting stuff done, making sure that he’s doing everything, making sure that our names are going to be put down for future tests and stuff, finding the exact genome of whatever it is that he [child] has that’s wrong, because they want to know exactly what’s wrong and stuff like that.” (F34)*
**For Policymakers**	
1. Funding for new and current programs and resources (utterances: 12)	*“We need more, more, more programs, more fully funded programs, uh that’s the key, that’s the key.” (F17)*
2. Need for leadership (utterances: 8)	*“I would tell them just to follow through on what they promise. If they say they’re going to look into something, look into something. If they, putting in a new bill that gives children more rights or something\, do it, you know.” (F6)*

Utterances refers to the total number of instances fathers spoke about a specific recommendation

The top three recommendations provided for healthcare and social service providers include: communicating authentically with families, listening to parents, and showing parents that they care. To fathers, communicating authentically with families involved communication that is honest, timely, informative, and understandable (see Table 2). It was also important to fathers that service providers show families that they care. Fathers expressed the need for more empathy (“We are more than a number”), understanding, and treating each family and child as unique. One father shared:

“We tried to let her know that this doesn’t work but every time she comes back with the same thing. Look at this book about bees. It’s like, well, its fine looking at a book but there’s a bee right there. It doesn’t work. Put a bee in a jar and try that. Every kid is unique.” (F22).

Finally, fathers emphasized the need for more support via funding and leadership. Fathers expressed that families of children with complex care needs are in dire need of funding for new and existing programs to increase the available spaces for children with CCN. Fathers also noted that funding was needed for financial assistance programs (e.g., income supplements) and programming to help primary caregivers with health issues relating to providing care (e.g., physical therapy, massage therapy):

“They should give more funding, especially for the primary caregivers. Mostly all the parents give up their careers, they stay home caring for their special need, and they have some physical needs too, like massage therapy. It’s, it’s hard.” (F27).

The need for leadership at the federal, provincial, and municipal levels was also stressed. Fathers highlighted that politicians need to take a leadership role in supporting families of children with CCN and follow through on their promises. Some fathers also stated there is not enough education for government leaders regarding the needs of these families (e.g., equipment needed, accessibility of community buildings, out-of-pocket expenses) and that this information should be provided to them.

“I don’t think there is enough education for our government leaders. They don’t really understand the whole concept. They understand kids have disabilities. Educate. Maybe they should walk in the shoes of a child with disabilities for a day or, you know, live a life of a family with a child with a disability that, you know, needs a standing frame and a walker and they’re only going to pay for one or the other. It’s – not everything’s covered, and some families just don’t have an income. When you have a child with special needs, you’re always paying for something and there’s no extra money, you know, and I don’t think people realize that.” (F21).

## Discussion

The purpose of this paper was to describe Canadian fathers’ experiences of caring for their child with complex care needs and to highlight recommendations they provided for other parents, service providers, and policymakers. To our knowledge, this is one of few Canadian studies to document fathers’ involvement in the life of their child with CCN beyond caregiving activities. Given the paucity of research on Canadian fathers’ experiences in this context, this study adds to the knowledge base regarding fathering a child with CCN. We identified one overarching theme (striving to be there for the child with CCN) and five supporting themes: 1) contributing to the parental team through various roles; 2) building accessibility through adaptation; 3) engaging in activities with the child; 4) expressing admiration and pride in their children; and 5) meaning making. Fathers in this study also provided recommendations for other parents in similar situations (e.g., make connections, ask for help), healthcare and social service providers (e.g., communicate authentically with families, listen to parents), and policymakers (e.g., the need for leadership and funding for programs) that can support fathers and parents of children with CCN in their caregiving role.

### Striving to be there for the child

Fathers in this study were committed to being there for their child with complex care needs through active engagement in activities and by being physically present with and for the child. The concept of “being there” has been discussed in past work detailing the caring experience of fathers of children with complex chronic conditions [61], terminal illnesses [62], disabilities [63, 64], as well as in the fathering literature about typically developing children [65, 66]. The enriching details we have provided of how fathers strived to be there for their child are of significance to service providers and programming designed to support fathers of children with CCN, especially those who are struggling to “be there” for their child.

In this study, we documented fathers’ active involvement in caring activities, expression of admiration and pride in their children and meaning making. All these activities have been documented in past research examining fathers’ experiences in this context [13, 25, 41, 42, 48, 61, 67–69]. Our study provides new insights about how fathers make participation in everyday life accessible and inclusive for their children. Fathers made adaptations and readjustments to physical spaces, obtained needed devices and tools, sought out accessible activities, and engaged in various activities with their child to this end. Fathers also noted they often encountered inaccessible physical environments (e.g., buildings, public facilities such as hockey arenas), transportation (e.g., personal vehicles), and urban design (e.g., snow-covered sidewalks in the winter, parking spots). In-depth exploration of the accessibility needs of families of children with CCN warrants further investigation.

### Fathers’ recommendations

When providing recommendations for other parents, fathers in this study highlighted the importance of obtaining social support (making new connections and nurturing old connections) and asking for help. This finding is consistent with past work that has found that fathers of children with CCN value support [41, 48, 70, 71]. More knowledge and understanding of support sources (e.g., peers, work) and support types (e.g., emotional, instrumental) that are important to fathers would be useful for interventions specifically targeting fathers’ well-being.

The recommendations provided by fathers for service providers (e.g., communicate authentically with families, listen to parents) highlight the importance fathers place on open communication and family-provider partnerships. The need for open communication and collaborative partnerships with service providers has been identified by families with complex needs [16, 72]. Research about fathers of children with life-limiting conditions also indicates that fathers often have difficulties communicating and forming relationships with service providers [16, 48] and believe health systems are biased towards engaging with mothers [48, 50, 73–75]. Thus, our findings highlight the importance of involving fathers (e.g., through open communication, active listening, sharing knowledge with both parents) to facilitate making them feel part of the family-provider partnership or team. Finally, fathers in this study identified the need for support from policymakers via funding for program development and sustainability to help with the child’s care (e.g., income supplements) and with the impacts of caregiving on parents. This finding is consistent with and supports previous work that has documented that these families: 1) encounter funding gaps as they navigate the health and social service systems [67, 76, 77]; 2) experience heavy financial burden [78, 79]; and 3) have unmet programming needs [44, 67, 77]. Fathers’ recommendations or solutions that are necessary to address the funding gaps warrant future exploration.

### Implications

In this study, fathers described their advocacy efforts as a struggle and a full-time job that involved fighting for necessary services and finding little support in the process (e.g., access to knowledge of programs/services available). Advocating for their child was at times stressful and emotionally taxing for parents. Healthcare and social service providers need to recognize the importance of validating parents’ experiences, working collaboratively with parents, supporting their advocacy efforts, and ensuring parents have the resources and information they need. In addition, healthcare provider responses perceived as excellent by fathers in this study (see Table 2) included listening to parents (e.g., trust/believe what they share, be open to feedback), communicating authentically with parental partners (e.g., being honest and forward, explaining medical terms, maintaining consistent and clear communication), and showing that they care (e.g., being understanding and empathetic, treating each family/child as unique). These factors are thus critical for forming collaborative relationships with fathers (and parents) and collaborative care teams in the clinical setting that can benefit both parents and their children.

Fathers in this study also worried about aging out of the pediatric complex care system and having to learn to navigate and plan in an unfamiliar adult health care system. Given that the adult healthcare and social service systems are fragmented [80, 81], our findings add to the call for complex care teams in the adult healthcare system that can provide the comprehensive and centralized services needed by these families. Young adults with CCN and their families want comprehensive and coordinated services developed with their input [82]. Given system changes can take time, service providers can advocate for policy development and funding to support continuity of care from pediatric to adult services. In addition, education of service providers about research-informed recommendations that can help these families, such as facilitating families’ early acquisition of knowledge of the adult healthcare system, connecting families to relevant resources, and initiating the transition process early [80, 81, 83], is warranted.

Our findings also have implications for those providing psychosocial support (e.g., therapists, counselors) to families of children with complex care needs. The majority of fathers in this study reported finding meaning in the experience of fathering a child with complex care needs. Fathers looked for and identified the good that their child brought to their lives. This highlights that once shock after diagnosis has subsided, growth is possible [84–86]. Encouraging healthy coping through meaning making may prove beneficial.

Finally, fathers reported making participation in everyday life possible for the child through accommodations (e.g., modifying physical environments, adapting or buying devices and needed technology). Fathers in this study had the financial means and the knowledge of resources to make these accommodations possible. However, many fathers noted the need for politicians’ leadership regarding accessibility and addressing barriers to ensure participation in every aspect of life is accessible to all. Accessible spaces (e.g., ensuring community buildings are accessible by wheelchair and mobility aid users, considering distance of parking to destination, addressing heavy doors) and funding programs (access to existing as well as new, inclusive programs) that allow children with CCN to thrive were identified by fathers as priority areas for policymakers.

### Strengths, limitations, and future research

To the best of our knowledge, this is the first study to use an ethnographic approach and arts-based methodologies to explore the experiences of Canadian fathers of children with complex care needs. Using ethnography and arts-based methods not only facilitated attaining a detailed understanding of fathers’ experiences but also provided creative ways for fathers to share their stories. Another strength is that our sample size was adequate and appropriate as theoretical saturation was achieved [87]. We have also used the Standards for Reporting Qualitative Research (SRQR) guidelines (see Supplemental information- S1 file) to enhance transparency and quality of reporting [88]. Finally, our sample consisted of fathers of children with a wide range of complex care needs. This facilitated data triangulation and enhanced the credibility and validity of our findings. Nonetheless, there are limitations. First, most fathers in our sample were of European descent and had an annual income of ≥ $60,000 (the median after-tax income of Canadian families and unattached individuals in 2021 was $68,400 [89]. Future work should aim for more diverse samples and examine the experiences of diverse fathers (e.g., ethnically diverse fathers, stepfathers, 2SLGBTQIA + fathers) as well as the role of intersectionality in fathers’ experiences. The intersection between pre-existing inequities and caregiving stressors and their impact on fathering needs to be better understood. Second, the majority of fathers in our study created meaning from the experience of fathering a child with CCN. Future research about fathers of children with CCN who have not created meaning out of their experience and about factors (e.g., religious/spiritual beliefs, education, culture) that influence the meaning-making process would be informative. Finally, our findings emerged from a larger study that sought to understand how parents of children with CCN participate in society. As such, parents spoke about their fathering experiences in that context. Future research should delve deeper into fathers’ support needs, adjustment and coping strategies, and resilience. Knowledge in this regard would be useful for parent-support programs and programing specifically designed for fathers.

## Conclusions

Through an ethnographic approach and the use of arts-based methodologies, we have painted a picture of Canadian fathers’ experiences of fathering a child with CCN. We provide evidence of fathers’ pivotal role in the care and development of their child and have identified actionable suggestions for parents, service providers, and policymakers that can serve to support these families. We have also highlighted areas necessitating research attention including the experiences of diverse fathers and the role of intersectionality in fathering children with CCN. By highlighting key areas of future exploration in this under-researched area, we hope to inform fathering research with this population to better understand and support fathers in this context.

### Supplementary Information

**Additional file 1.**

## Data Availability

The datasets used and/or analysed during the current study are available from the corresponding author on reasonable request.

## Abbreviations

CCN: Complex care needs
FAC: Family advisory committee
